# Father-to-daughter transmission in late-onset OTC deficiency: an underestimated mechanism of inheritance of an X-linked disease

**DOI:** 10.1186/s13023-023-02997-8

**Published:** 2024-01-02

**Authors:** Barbara Siri, Giorgia Olivieri, Francesca Romana Lepri, Martin Poms, Bianca Maria Goffredo, Anna Commone, Antonio Novelli, Johannes Häberle, Carlo Dionisi-Vici

**Affiliations:** 1 Division of Metabolic Diseases and Hepatology, Bambino Gesù Children’s Hospital, IRCCS, Piazza S. Onofrio 4, 00165 Rome, Italy; 2 Department of Paediatrics, Città della Salute e della Scienza, OIRM, University of Turin, Turin, Italy; 3 Translational Cytogenomics Research Unit, Laboratory of Medical Genetics, Bambino Gesù Children’s Hospital, IRCCS, Rome, Italy; 4 Division of Clinical Chemistry and Biochemistry and Children’s Research Center, University Children’s Hospital Zurich, University of Zurich, Zurich, Switzerland; 5 Division of Metabolism and Metabolic Diseases Research Unit, Bambino Gesù Children’s Hospital, IRCCS, Rome, Italy; 6 Division of Metabolism and Children’s Research Center, University Children’s Hospital Zurich, University of Zurich, Zurich, Switzerland

**Keywords:** Ornithine transcarbamylase deficiency (OTCD), X-Linked, Father-to-daughter transmission, Hyperammonemia

## Abstract

**Background:**

Ornithine Transcarbamylase Deficiency (OTCD) is an X-linked urea cycle disorder characterized by acute hyperammonemic episodes. Hemizygous males are usually affected by a severe/fatal neonatal-onset form or, less frequently, by a late-onset form with milder disease course, depending on the residual enzymatic activity. Hyperammonemia can occur any time during life and patients could remain non- or mis-diagnosed due to unspecific symptoms. In heterozygous females, clinical presentation varies based on the extent of X chromosome inactivation. Maternal transmission in X-linked disease is the rule, but in late-onset OTCD, due to the milder phenotype of affected males, paternal transmission to the females is possible. So far, father-to-daughter transmission of OTCD has been reported only in 4 Japanese families.

**Results:**

We identified in 2 Caucasian families, paternal transmission of late-onset OTCD with severe/fatal outcome in affected males and 1 heterozygous female. Furthermore, we have reassessed the pedigrees of other published reports in 7 additional families with evidence of father-to-daughter inheritance of OTCD, identifying and listing the family members for which this transmission occurred.

**Conclusions:**

Our study highlights how the diagnosis and pedigree analysis of late-onset OTCD may represent a real challenge for clinicians. Therefore, the occurrence of paternal transmission in OTCD should not be underestimated, due to the relevant implications for disease inheritance and risk of recurrence.

**Supplementary Information:**

The online version contains supplementary material available at 10.1186/s13023-023-02997-8.

## Introduction

Ornithine Transcarbamylase Deficiency (OTCD) (OMIM# 311250), the most common of urea cycle defects (UCDs) with an estimated incidence of 1 in 66.000–70.000 [1, 2], is caused by mutations in *OTC* gene mapping on Xp11.4 [3, 4]. OTC, mainly expressed in liver and intestine, catalyzes the reaction between carbamoyl phosphate and ornithine to form citrulline and phosphate, initiating the ammonia detoxification process that generates arginine and urea [5]. Deficiency of this enzyme results in impaired ureagenesis and build-up of ammonia in the bloodstream [6].

Among urea cycle defects, OTCD is the only inherited as an X-linked recessive trait. Hemizygous males are usually affected by a very severe neonatal-onset form or, less frequently, by a late-onset form, with milder disease course, mainly depending on the OTC residual enzymatic activity [6, 7]. Symptoms of this late-onset form include recurrent vomiting, protein aversion, unexplained hepatopathy, intermittent neurological and psychiatric symptoms with emotional or personality changes, up to an overt encephalopathy with seizures and coma. Patients may remain asymptomatic even for long periods of time, or may present chronic disease course with intermittent episodes of hyperammonemia which are often triggered by intercurrent illnesses, dietary changes, drugs exposure (e.g. valproate, asparaginase), catabolic stressors or prolonged fasting [7–11].

In females, clinical presentation of OTCD varies widely [12, 13], with most heterozygous females being asymptomatic, but at least 20% of female carriers manifesting some degree of disease expression [14], mainly based on the extent of mutant X chromosome inactivation (XCI) [15, 16].

Beside *de novo* mutation, which may occur in a non-negligible number of cases, maternal inheritance is considered a paradigm in X-linked disorders, however paternal transmission may occur as rarely seen in some neurological and neuromuscular diseases [17–19]. In late-onset OTCD, paternal transmission has been so far reported only in a few Japanese families carrying two recurrent variants, c.119G > A (p.Arg40His) and c.163T > G (p.Tyr55Asp) [11, 20, 21].

Herein, we describe a detailed molecular and functional study in two Caucasian families affected by late-onset OTCD with documented father-to-daughter transmission.

## Patients and methods

### Family 1

**Fig. 1:**
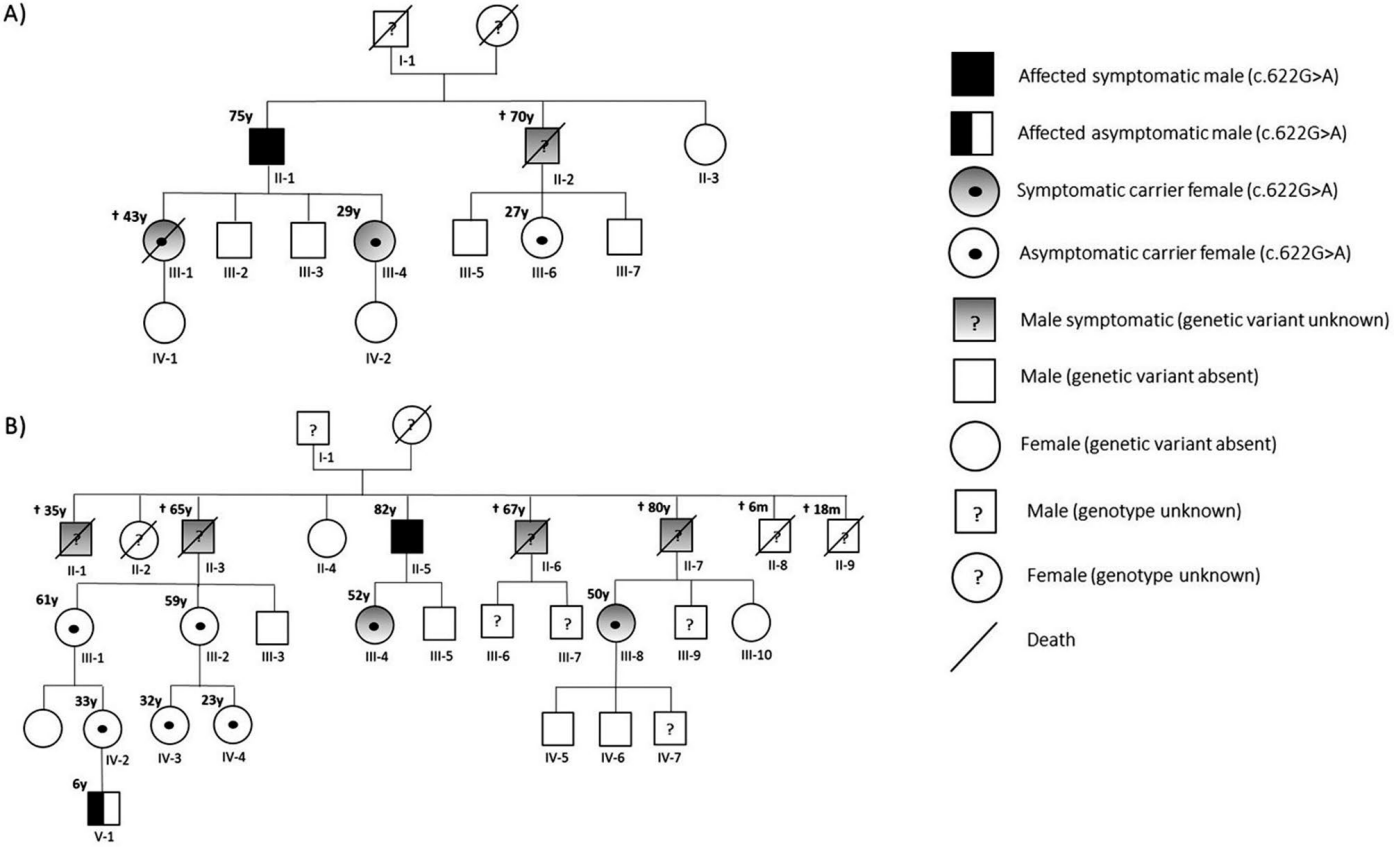
Pedigree of two Italian families with paternal transmission of OTC deficiency: Panel (A) Family 1, Panel (B) Family 2

The pedigree and metabolic profiles of Family 1 are reported in Fig. 1 and Table 1, respectively. A 43-year-old woman (III-1) was admitted to the hospital for altered mental status with confusion and reduced alertness after treatment with steroids and nonsteroidal anti-inflammatory drugs (NSAID) for arthritis. During the following days, the clinical picture rapidly worsened up to status epilepticus and coma. Laboratory investigations revealed a severe hyperammonemia (ammonia 805 µmol/L, normal value (n.v.) < 50) with brain edema at CT scan. Poor response to ammonia scavengers and hemodialysis treatment resulted in death of patient after few days. A post-mortem metabolic assessment revealed elevated plasma glutamine (1295 µmol/L, n.v. 200–800), despite normal citrulline (21 µmol/L, n.v. 10–35) and arginine (63 µmol/L, n.v. 30–90) and increased urinary orotic acid excretion (208 mmol/mol creatinine, n.v. 0.1–10), leading to the diagnostic suspicion of OTCD. Past medical history was characterized by protein-food aversion and episodes of irritability and aggressive behavior. Starting from the age of 36, these episodes worsened with headache, confusion, decreased alertness and vomiting rarely requiring hospitalization and episodes were preceded by corticosteroids and/or NSAIDs assumption. The proband’ sister (III-4), a 29-year-old woman, had a negative personal history except for occasional episodes of nausea, asthenia, migraine, and irritability. Her metabolic profile showed normal ammonia and plasma amino acids, absent orotic aciduria also after an allopurinol loading test. The proband’s father (II-1), a 75years-old male, showed recurrent episodes of migraine and irritability but normal ammonia and plasma amino acid profile. A paternal uncle (II-2) with a positive history of chronic heart disease, type II diabetes, chondrosarcoma, and chronic kidney failure, at 67 years developed an episode of migraine followed by mild dysphasia and weakness of left face with rapid progression to aphasia and weakness to right upper limb. Brain CT and MRI revealed left subdural hematoma and a globally slow and low voltage electrical activity at EEG. Laboratory workup showed elevated ammonia (up to 210 µmol/L) with normal liver function tests, but steatosis at abdominal ultrasound. The clinical picture and laboratory profile showed a good response to mannitol, steroids, and ammonia scavengers. He died of prostatic cancer at 70 years. He never reported protein-food aversion. His daughter, a 27-year-old woman (III-6), showed a clinical picture like her cousin (III-4) with a normal metabolic profile.

**Table 1:** Biochemical profile, treatment, and dietary regimen of OTC deficient patients in Family 1 and Family 2

	Sex	Age (years)	Symptoms	Blood ammonia (n.v.<50 µmol/L)	Plasma glutamine (n.v. 200–800 µmol/L)	Plasma citrulline (n.v. 10–35 µmol/L)	Plasma arginine (n.v. 30–90 µmol/L)	Urinary orotic acid (0.1–10 mol/mmol creat)	Therapy	Protein restricted diet
						**Family 1**				
**II-1**	M	73	migraine, irritabilty	22	714	18	47	0.5	arginine	yes
**III-1**	F	43	coma, death	805	1295	21	63	208	no	no
**III-4**	F	27	nausea, migraine, irritabilty	22	712	28	43	0.5	arginine	yes
**III-6**	F	29	nausea, migraine, irritabilty	22	603	10	22	0.3	arginine	yes
						**Family 2**				
**II-5**	M	74	confusion, abnormal behaviour	76	1279	14	50	3.0	arginine, GPB	yes
**III- 1**	F	58	no	6	674	25	74	not available	no	no
**III-2**	F	56	no	7.7	790	36	45	not available	no	no
**III-4**	F	51	migraine	26	806	24	54	0.8	arginine	yes
**III-8**	F	50	migraine, vomiting	26	876	26	48	0.4	arginine	yes
**IV-2**	F	31	no	14	753	29	84	not available	no	no
**IV-3**	F	31	no	13	658	13	34	not available	no	no
**IV-4**	F	23	no	22	787	26	80	not available	no	no
**V-3**	M	4	no	26	598	21	47	0.6	no	no

**Abbreviations**: M: male; F: female; GBP: glycerol phenylbutyrate

Protein restricted diet (0.5–0.9 g/kg/die) and L-Arginine supplementation (53–120 mg/kg/die) has been prescribed in the father (II-1), sister (III-4) and cousin (III-6) of the proband with good clinical response.

### Family 2

The pedigree and metabolic profiles of Family 2 are reported in Fig. 1 and in Table 1, respectively. A 52-year-old woman (III-4), daughter of non-consanguineous parents, was referred to us because of chronic headache and protein aversion. Her maximum ammonia level during symptoms was 107 µmol/L requiring ammonium scavenger therapy and protein restricted diet. Her father (II-5), an 82-year-old, experienced occasional episodes of confusion associated with abnormal behaviour. In symptom-free period his maximun ammonia level was 76 µmol/L with elevated glutamine (1279 µmol/L) and normal citrulline (14 µmol/L). Six paternal uncles had died, four of them (II-1, II-3, II-6 and II-7) between the ages of 30 and 80 years after acute episodes characterized by impairment of consciousness, rapidly developing coma and death in less than 12 h. Before these fatal episodes, they never experienced symptoms. In these subjects, laboratory work-up and imaging studies respectively revealed hyperammonemia and cerebral edema at CT scan. The potential triggers for these episodes of acute decompensation were not clear, although one uncle (II-6) had previous NSAIDs therapy and another two uncles (II-3 and II-7) were treated with diazepam for psychomotor agitation. Furthermore, two additional parental uncles (II-8 and II-9) and a paternal aunt (II-2) of the proband died between the ages 6 and 18 months from unexplained causes. A female 50-year-old cousin of the proband (III-8), whose father died at 31 years for hyperammonemic encephalopathy (II-7), also presented a history of migraine, vomiting associated with behavioural changes, with glutamine at the upper limit (876 µmol/L) and normal citrulline and arginine without orotic aciduria. The family history appeared compatible with OTC deficiency. The proband and her symptomatic cousin (III-4 and III-8) started protein restricted diet (0.5 g/kg/die) and arginine supplementation (69 − 66 mg/kg/die); while the proband’s father (II-5) was given in addition glycerol phenylbutyrate (GBP) for persistently elevated glutamine levels, with good clinical and biochemical responses. The analysis of pedigrees in the family also allowed the identification of 5 asymptomatic females, proband’s cousins (III-1; III-2; IV-2; IV-3; IV-4) and a 6-years-old asymptomatic male (V-1) all with normal amino acid profiles and on a free protein diet.

## Methods

Informed consent was obtained from patients or their parents and the data were obtained in accordance with the Helsinki Declaration of 1964 as revised in 2000.

DNA was isolated from peripheral blood cells and molecular investigations with Next-Generation Sequencing (NGS) (NovaSeq6000 Illumina) of *OTC* were carried out. Interpretation and classification of identified variants were performed following American College of Medical Genetics (ACMG) recommendations for variant classification [22, 23]. Co-segregation analyses among families were conducted. Analysis of ureagenesis function was performed in three patients, one of Family 1 (II-1) and two of Family 2 (II-5, III-4) to characterized the in vivo function of the urea cycle (ClinicalTrials.gov Identifier: NCT05671666). This ureagenesis test allows to evaluate the isotopic enrichment of plasma urea and urea cycle-related metabolites at six different time points during two hours after ingestion of a small dose of a [^15^N] labelled tracer using a modification of a published protocol [24] that was developed building up on the method initially described in 1996 with the same tracer but at that time less sensitive technology [25] The ureagenesis quantification assay developed by Yudkoff and colleagues exploits the fast absorbance of an oral tracer in the small intestine and its almost immediate transport to periportal hepatocytes at high quantity due to the first pass in liver.

Descriptive statistics were presented as median with Interquartile Ranges (IQR), 25th and 75th values (mean; IQR 25th-75th), for non-normally distributed values, using GraphPad Prism 8 (GraphPad Software, Inc., San Diego, CA).

## Results

### Molecular investigations

Pedigree and molecular analysis with number and type of transmission are reported in Fig. 1; Table 2. In Family 1, a missense c.622G > A (p.Ala208Thr) variant in exon 6 of *OTC* was identified in the proband (III-1). Segregation analysis of all family members showed that the c.622G > A change was inherited from the father (II-1). The variant was also detected in the proband’s sister (III-4) and in a female cousin (III-6), whose father presented late-onset (67-years) hyperammoniemic encephalopathy, indicating the paternal inheritance from the two hemizygous male siblings (II-1, II-2). In Family 2, *OTC* gene sequencing in proband (III-4) revealed the presence of a missense variant, c.1033T > C (p.Tyr345His), in exon 10 of *OTC*. Segregation studies showed that this variant was inherited from her father (II-5). The extension of molecular investigations to other family members revealed the presence of c.1033T > C change in three others heterozygous females (III-1, III-2, and III-8), who inherited the variant from their fathers (II-3 and II-7) who died from hyperammonemic encephalopathy respectively at 65 and 80 years. The variant was also maternally transmitted to five asymptomatic 2nd and 3rd degree cousins of the proband, three females (IV-2, IV-3, IV-4) and one male (V-1).

**Table 2:** Father-to-daughter transmission of OTC deficiency

Reference	Variant	Liver OCT activity	Ethnicity	Affected males	Heterozygous females	Father-to-daughter transmission	Mother-to-daughter transmission	Mother-to-son transmission
[23]	c.119G > A (p.Arg40His)	< 3%	Japanese	II-10 (48y, fatal HA) II-4 (48y, fatal HA) IV- 2 (symptomatic)	I-2, II-12 (no clinical information) III-4 and III-5 (asymptomatic)	II-4 > III-4 II-4 > III-5	I-2 > II-12	I-2 > II-4 I-2 > II-10 III-5 > IV-2
	c.119G > A (p.Arg40His)		Japanese	I-1 (46y, fatal HA)	II-1 (17y, motor paresis, ID)	I-1 > II-1		
	c.119G > A (p.Arg40His)	3.4%	Japanese	III-1 (32y, fatal HA) III-4 (21y, symptomatic)	I-2, II-2, II-4 (no clinical information) IV-1 (asymptomatic)	III-4 > IV-1	I-2 > II-2 I-2 > II-4	II-2 > III-1 II-4 > III-4
	c.163T > G (p.Tyr55Asp)	< 2%	Japanese	I-1 (46y, fatal HA) (one individual hepatitis, 51y, one individual hepatoma, 68y)	II-1 (orotic aciduria after protein load)	I-1 > II-1	no	no
[23]	c.622G > A (p.Ala208Thr)		American	I-1 (52y, fatal HA) III-1 (NH_3_ 182) II-2 (NH_3_ 265)	II-1 (no clinical information)	I-1 > II-1		II-1 > III-1 II-1 > III-2
[23]	c.622G > A (p.Ala208Thr)		Italian	I-1 (66y, HA)	II-I (no clinical information)	I-1 > II-1	no	no
[23]	c.622G > A (p.Ala208Thr)		Netherlands	IV-1 (67y, fatal HA) VI-4, V-6, V-8	III-6, IV-4, IV-5, V-3, V-4 (no clinical information)	IV-1 > V-3 IV-1 > V-4 II-3 > III-6		V-4 > VI-4 IV-4 > V-6 IV-4 > V-8
[23]	c.622G > A (p.Ala208Thr)	3%	Polish	I-9 (asymptomatic) IV-14, V-4 (orotic aciduria after allopurinol test)	IV-7 (14y, fatal HA) II-2, II-4, II-9, III-7, III-9, III-18, IV-6 (asymptomatic) (IV-6: orotic aciduria)	I-9 > II-2 I-9 > II-4 I-9 > II-9	II-4 > III-7 II-4 > III-9 II-4 > III-15 II-4 > III-18 II-6 > III-17 II-6 > III-18 II-9 > III-19 III-7 > IV-6 III-9 > IV-9	III-7 > IV-7 III-18 > IV-14 IV-6 > V-4
[23]	c.622G > A (p.Ala208Thr)	4%	Netherlands	I-1, III-1, IV-3, IV-9 (asymptomatic) III-1, IV-3, IV-9 (orotic aciduria)	IV-5 (10y, fatal HA) II-1, II-4, III-2, III-3, III-4, IV-6 (asymptomatic) III-2 (orotic aciduria)	I-1 > II-2 I-1 > II-4	II-1 > III-2 II-1 > III-3 III-3 > IV-6	II-1 > III-1 III-2 > IV-3 III-3 > IV-5 III-4 > IV-9
[23]	c.622G > A (p.Ala208Thr)		Japanese	IV-1 (6y, fatal HA)	II-5 (68y, fatal HA) III-2, III-3, IV-3 (no clinical information)	II-5 > III-2 II-5 > III-3	III-2 > IV-3	III-2 > IV-1
[23]	c.622G > A (p.Ala208Thr)		French	III-1, III-2 (no clinical information)	I-1 (no clinical information) II-2 (49y, severe HA) III-3 (21y, asymptomatic)	II-2 > III-3	suspected I-1 > II-2	II-1 > III-1 II-1 > III-2
**Family 1**	c.622G > A (p.Ala208Thr)		Italian	II-1 (migraine, aphasia, weakness), II-2 (67y, HA)	III-1 (43y, fatal HA) III-4, III-6 (migraine, irritability, asthenia)	II-1 > III-1 II-1 > III-4 II-2 > III-6	no	no
**Family 2**	c.1033T > C (p.Tyr345His)		Italian	II-5 (confusion, abnormal behaviour)	III-1, III-2 (asymptomatic) III-6 and III-10 (migraine, asthenia, irritability)	II-5 > III-6 II-7 > III-10	III-1 > IV-2 III-2 > IV-3 III-2 > IV-4	IV-2 > V-3

**Abbreviations**: HA: hyperammonemia; ID: intellectual disability

### Functional studies

Ureagenesis capacity was assessed in the father’s proband (II-1) in Family 1 and in the proband and her father (III-4 and II-5) in Family 2. Comparing whether the isotope tracer was cleared through the urea cycle, it was possible to determine ureagenesis capacity compared to control values.

Both males of Family 1 (II-1) and 2 (II-5) showed a mild impairment of their ureagenesis function when compared to 21 healthy controls while total urea concentrations were in the normal range and citrulline was low.

The mildly symptomatic female (F2 III-4) showed a borderline normal ureagenesis capacity, again when compared to 21 healthy controls, associated with increased glutamine, but normal urea and citrulline.

## Discussion

We report on two families showing paternal transmission of late-onset OTCD with severe fatal outcome in both affected males and heterozygous females.

Father-to-daughter transmission in OTCD represents a non-conventional inheritance pattern. However, a late-onset phenotype in affected males reaching reproductive age may allow the paternal transmission of *OTC* variants to their female progeny. Of note, another extremely rare mechanism of paternal OTC transmission is represented by germinal mosaicisms with few case reports in the literature [26–28].

So far, documented father-to-daughter OTCD transmission have been only highlighted in four Japanese families carrying two recurrent variants, c.119G > A (p.Arg40His) and c.163T > G (p.Tyr55Asp) [11, 20, 21] (Table 2).

The paternal transmission of c.119G > A (p.Arg40His) variant was identified in three families with two affected males presenting fatal hyperammonemic episodes at 46 and 48 years, and one 21-year-old male subject with a milder OTCD phenotype. In these three families, four father-to-daughter transmissions occurred in three asymptomatic females and one 17-year-old female presenting motor paresis and intellectual disability [21].

Another recurrent variant, c.163T > G (p.Tyr55Asp), was reported in a male patient with an OTC activity of < 2% presenting a fatal encephalopathy at the age of 46 years. Pedigree analysis identified a paternal transmission of this variant to his asymptomatic daughter [11, 20, 21].

Molecular investigation in our families identified in Family 1 the missense c.622G > A (p.Ala208Thr) variant and in Family 2 the missense c.1033T > C (p.Tyr345His) change, and pedigree analysis revealed paternal transmission of both variants.

The c.622G > A (p.Ala208Thr) variant has been associated with late-onset OTCD phenotype, however it has also been reported to cause severe disease in childhood in males [16, 29–40]. The c.622G > A (p.Ala208Thr) change is located in ornithine binding domain of OTC and, therefore, variants substitution could results in deleterious effect of protein function. This variant has been classified as pathogenic by ACMG and not observed in large population cohorts (gnomAD).

Reassessment of the existing literature shows the prior report of seven families showing paternal inheritance of the c.622G > A (p.Ala208Thr) substitution [29–35] (Table 2) with wide intra- and inter-familial phenotypic variability in terms of disease onset and severity among male carriers of this substitution.

The seven males carrying the mutated alleles showed a wide phenotypic expression, ranging from fatal/severe hyperammonemic events [29–31, 34, 35] to a complete absence of clinical signs [32, 33]. In one patient, who had died at 10 years for hyperammonemic encephalopathy, the residual OTC activity was 4% in the liver [30]. The phenotype of reported females receiving the muted OTCD allele from their fathers was generally mild or asymptomatic.

In Family 1, the pedigree analysis identified the paternal transmission of the c.622G > A (p.Ala208Thr) change from two brothers with mild OTCD to their three daughters with a clinical phenotype ranging from a severe fatal hyperammonemic episode at 43 years in one patient to milder symptoms in the other heterozygous females. Combining the reported cases with Family 1, the c.622G > A (p.Ala208Thr) variant is usually associated with a late-onset OTCD, but fatal outcome could present at any age, from childhood to adulthood, both in male and female patients.

The c.1033T > C (p.Tyr345His) variant identified in Family 2, has been reported only in one male with late-onset OTCD [41]. This variant has been classified as pathogenic by ACMG and has not been observed in large populations cohorts (gnomAD). Furthermore, two variants at the same amino acid position but with different amino acid changes, c.1033 A > G (p.Tyr345Cys) and c.1033T > G (p.Tyr345Asp), have been identified in a male with neonatal onset OTCD and in a 3-months-old female with fatal hyperammonemic encephalopathy [42–44].

The molecular analysis of the large pedigree in Family 2 allowed to identify four father-to-daughter, three mother-to-daughter and one mother-to-son transmissions. The clinical presentation showed a wide disease spectrum: from fatal outcomes and different age of disease presentation in males (30–80 years) to oligo-asymptomatic heterozygous female patients.

Functional ureagenesis studies in both families confirmed a mild impairment of ureagenesis in affected males and a normal function in a female carrier for both mutations.

## Conclusions

This study highlights how the diagnosis and pedigree analysis of late-onset OTCD may sometimes represent a real challenge for clinicians [45, 46]. As seen in our families and in other reports, the occurrence of father-to-daughter transmission in OTCD should not be underestimated due to the relevant implications for genetic counseling in terms of mechanism of disease inheritance and risk of recurrence.

### Electronic supplementary material

Below is the link to the electronic supplementary material.

Supplementary Material 1

## Data Availability

All data generated and analyzed during this study are included in this published article.

## Abbreviations

OTCD: Ornithine Transcarbamylase Deficiency
UCD: urea cycle defect
XCI: X chromosome inactivation
NSAID: non-steroidal anti-inflammatory drugs
NGS: Next-Generation Sequencing
ACMG: American College of Medical Genetics
IQR: Interquartile Ranges
