# Supplementary material for: Father-to-daughter transmission in late-onset OTC deficiency: an underestimated mechanism of inheritance of an X-linked disease
Source: Orphanet J Rare Dis. 2024 Jan 2;19:3. doi: 10.1186/s13023-023-02997-8 (PMC10763478; doi:10.1186/s13023-023-02997-8)
Supplement: Supplementary file 1 — Supplementary Material 1 [file 13023_2023_2997_MOESM1_ESM.docx]

**Highlights**

- Father-to-daughter transmission in OTC is an underestimated pattern of inheritance
- Severe hyperammoniemic episodes occur in late-onset OTC adult males and females
- Paternal OTC inheritance is relevant for genetic counselling and risk of recurrence
